# Enhanced hemocompatibility and rapid magnetic anastomosis of electrospun small-diameter artificial vascular grafts

**DOI:** 10.3389/fbioe.2024.1331078

**Published:** 2024-01-24

**Authors:** Peng Liu, Xin Liu, Lifei Yang, Yerong Qian, Qiang Lu, Aihua Shi, Shasha Wei, Xufeng Zhang, Yi Lv, Junxi Xiang

**Affiliations:** ^1^ Center for Regenerative and Reconstructive Medicine, Med-X Institute, The First Affiliated Hospital of Xi’an Jiaotong University, Xi’an, Shaanxi, China; ^2^ National Local Joint Engineering Research Center for Precision Surgery and Regenerative Medicine, The First Affiliated Hospital of Xi’an Jiaotong University, Xi’an, Shaanxi, China; ^3^ Department of Graduate School, Xi’an Medical University, Xi’an, Shaanxi, China; ^4^ Department of Hepatobiliary Surgery, The First Affiliated Hospital of Xi’an Jiaotong University, Xi’an, Shaanxi, China; ^5^ Department of Geriatric Surgery, The First Affiliated Hospital of Xi’an Jiaotong University, Xi’an, China

**Keywords:** coaxial electrospinning, magnetic anastomosis, small-diameter vascular grafts, hemocompatibility, sutureless

## Abstract

**Background:** Small-diameter (<6 mm) artificial vascular grafts (AVGs) are urgently required in vessel reconstructive surgery but constrained by suboptimal hemocompatibility and the complexity of anastomotic procedures. This study introduces coaxial electrospinning and magnetic anastomosis techniques to improve graft performance.

**Methods:** Bilayer poly(lactide-co-caprolactone) (PLCL) grafts were fabricated by coaxial electrospinning to encapsulate heparin in the inner layer for anticoagulation. Magnetic rings were embedded at both ends of the nanofiber conduit to construct a magnetic anastomosis small-diameter AVG. Material properties were characterized by micromorphology, fourier transform infrared (FTIR) spectra, mechanical tests, *in vitro* heparin release and hemocompatibility. *In vivo* performance was evaluated in a rabbit model of inferior vena cava replacement.

**Results:** Coaxial electrospinning produced PLCL/heparin grafts with sustained heparin release, lower platelet adhesion, prolonged clotting times, higher Young’s modulus and tensile strength *versus* PLCL grafts. Magnetic anastomosis was significantly faster than suturing (3.65 ± 0.83 vs. 20.32 ± 3.45 min, *p* < 0.001) and with higher success rate (100% vs. 80%). Furthermore, magnetic AVG had higher short-term patency (2 days: 100% vs. 60%; 7 days: 40% vs. 0%) but similar long-term occlusion as sutured grafts.

**Conclusion:** Coaxial electrospinning improved hemocompatibility and magnetic anastomosis enhanced implantability of small-diameter AVG. Short-term patency was excellent, but further optimization of anticoagulation is needed for long-term patency. This combinatorial approach holds promise for vascular graft engineering.

## 1 Introduction

Vascular diseases are major causes of morbidity and mortality globally (Feng et al., 2022). Bypass surgery or vascular reconstruction is the only course of correction in some cases (Seifu et al., 2013; Johnson et al., 2019). Artificial vascular grafts (AVGs) composed of polyethylene terephthalate, polytetrafluoroethylene and polyurethane can effectively replace blood vessels with large diameters (Anderson et al., 2018; Leal et al., 2021; Popa et al., 2022). However, these materials have poor patency and frequent thrombosis when replacing vessels <6 mm (Baguneid et al., 2011; Eilenberg et al., 2020). To address the challenges of small-diameter AVGs, innovations like coaxial electrospinning, decellularization, freeze-drying and 3D printing have been developed (Darie-Niță et al., 2022). Coaxial electrospinning in particular shows promise for nanofibrous vascular grafts (Laktionov et al., 2014; Yin et al., 2017; Liu et al., 2022). This method enables the incorporation of anticoagulant agents (Huang et al., 2013; Kuang et al., 2018), within the core-shell structure of the nanofibers, thus enhancing their antithrombotic properties.

However, electrospun AVGs have limited compliance compared to native vessels (Faturechi et al., 2019a). This makes the anastomosis difficult, increases the leakage risk at the anastomosis site, and impairs patency (Tamimi et al., 2019; Jeong et al., 2020). Magnetic anastomosis enables sutureless magnetic vessel connections, reducing leakage and hemorrhage risks compared to suturing (Kubo et al., 2018; Isozaki et al., 2020). Therefore, we hypothesize this technique can address the compliance mismatch problem in small-diameter AVGs.

**FIGURE 1:**
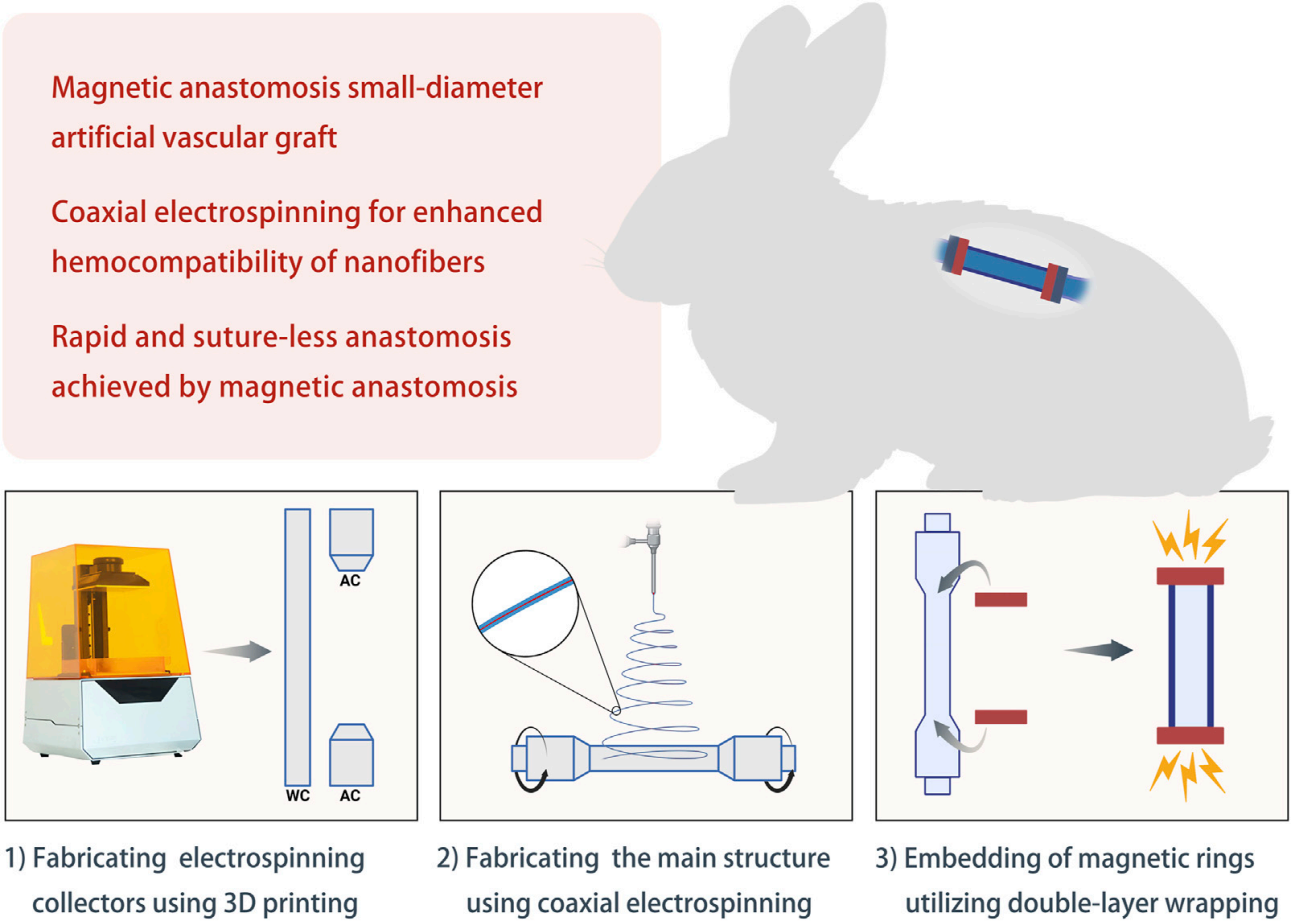
Schematic diagram of the study design Abbreviations: WC, working collector; AC, auxiliary collector.

In this study, we develop and apply a small-diameter AVG using coaxial electrospinning and magnetic anastomosis. We first fabricated polymer-derived nanofibers with enhanced hemocompatibility by applying coaxial electrospinning. We then developed a small-diameter AVG integrating magnetic anastomosis. Finally, we implanted the AVG in a rabbit model to evaluate its *in vivo* performance (Figure 1).

## 2 Materials and methods

### 2.1 Materials

Poly (l-lactic acid-co-ε-caprolactone) (PLCL, LLA/CL = 1/1, 15 kDa, Jinan Daigang Biomaterial Co., Ltd., China) was dissolved in 1,1,1,3,3,3-hexafluoro-2-propanol (HFIP, Aladdin, United States) at 200 mg/mL. Heparin sodium (13 kDa, Runjie Medicine Chemical, China) was dissolved in saline at a concentration of 100 mg/mL. NdFeB magnetic rings were procured from Jiujiu High-Tech Magnetic Materials Ltd. in China, featuring an inner diameter of 3.3 mm, an outer diameter of 4.9 mm, and a thickness of 1.5 mm.

### 2.2 Artificial vascular grafts preparation

**FIGURE 2:**
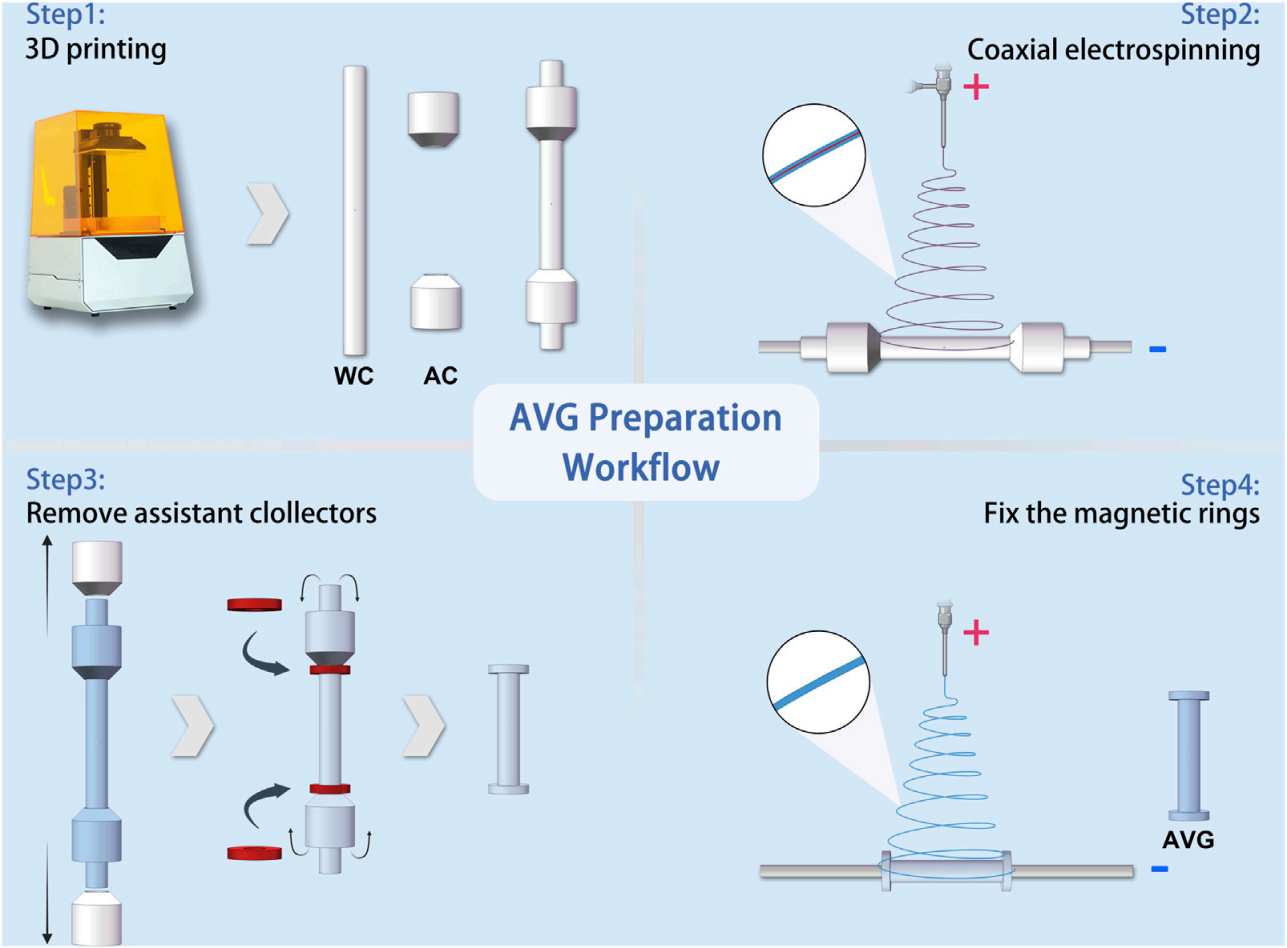
Schematic workflow of artificial vascular graft preparation Abbreviations: WC, working collector; AC, auxiliary collector.

AVGs were prepared as previously described (Liu et al., 2022) with modifications. As shown in Figure 2, a 3D printer (Xiao Fang ONE, Exquisitely 3D, China) was used to fabricate electrospun collectors. The collectors were classified into two distinct types based on their functions: the working collector (WC) and the auxiliary collector (AC).

An electrospinning machine (ET-2535H, Beijing Yongkang Leye Technology Development Co., Ltd.) was used to create the electrospun fibers. The machine had two syringe pumps to deliver solutions to a coaxial needle (22G+17G, Hefei Sipin Technology Co., Ltd.). The outer needle dispensed the outer solution at 2 mL/h, while the inner needle dispensed the inner solution at 0.2 mL/h. A high-voltage power supply generated 18 kV positive voltage with a 15 cm distance between the spinneret and collector. The coaxial needle had an inner diameter of 22G and outer diameter of 17G. For comparison, single-spinneret electrospinning was done under the same conditions, except with a 9 kV voltage and a 22G single-channel needle.

The magnetic anastomosis AVG (mag-AVG) was fabricated using a double layer wrapping method. Figure 2 illustrates the workflow of the process, which involved coaxial electrospinning on one working collector (WC) and two auxiliary collectors (ACs) at a speed of 75 rpm, forming an inner layer with a bell-mouth structure. Afterward, the ACs were removed, and the magnetic rings were inserted. Subsequently, the magnetic rings were wrapped with the bell-mouth structure. Finally, a second layer of electrospinning was performed on the WC at a speed of 50 rpm for 15 min. In contrast, the suture anastomosis AVG (suture-AVG) was fabricated using a similar method, except that no magnetic rings were embed.

### 2.3 Micromorphology

The surface/cross-section morphologies were measured via a scanning electron microscope (SEM, TM-1000, Japan). The core-shell structures were confirmed by electrospinning fibers onto a carbon-coated copper mesh and examining them with transmission electron microscopy (TEM, H-7650, Japan) at 80 kV.

### 2.4 Fourier transform infrared spectroscopy

IR spectra (PLCL fibers, PLCL/heparin fibers, and heparin) were acquired using a Thermo Nicolet iS50 FTIR infrared spectrometer. Each spectrum was obtained by averaging 64 scans, and the wavenumber range spanned from 400 to 4,000 cm^−1^.

### 2.5 Mechanical properties

To assess the mechanical properties of the AVGs, we conducted tensile testing utilizing a universal testing machine (CMT8502, New Sans Test Technical Company, China). The specimens were loaded at a constant strain rate of 10 mm/min until rupture.

The burst pressure of the artificial blood vessels was determined by a vascular anastomosis experimental system (YZ-08, Xi’an Magnat Medical Technical Company, China).

Following ANSI 7198, compliance measurements were made on ∼6 cm AVG segments by a vascular anastomosis experimental system, as described in 2.5. The external diameter was measured from digital images recorded. Compliance was calculated and reported as % per 100 mmHg as follows (Konig et al., 2009):

$$C=\frac{\left(R_{1}-R_{0}\right)/R_{0}}{P_{outlet}-P_{inlet}}\times 10^{4}$$

where C is compliance (%), R_0_ is the original graft diameter, R_1_ is the changed graft diameter, P_inlet_ is the inlet pressure and P_outlet_ is the outlet pressure.

### 2.6 *In vitro* release of heparin

To assess heparin release, 300 mg samples were immersed in 10 mL PBS (pH 7.4) at 37°C. At specific time intervals, 1 mL of the solution was extracted and replaced with fresh PBS to measure heparin concentration. Heparin test kits (G-CLONE Biotechnology Co., Ltd.) evaluated the initial heparin loading and release, with all measurements done in triplicate.

### 2.7 Hemocompatibility analysis

To investigate platelet adhesion, fresh anticoagulated rabbit blood was centrifuged to obtain platelet-rich plasma (PRP) (Liu et al., 2022). A 1.0 × 1.0 cm^2^ sample was immersed in PRP and incubated at 37°C for 1 h. The morphology and number of platelets adhered to the electrospun membrane were observed and quantified using SEM.

The hemolysis test began by collecting freshly anticoagulated rabbit blood and diluting it with saline. A 1 cm × 1 cm electrospun membrane was washed and incubated in saline at 37°C for 30 min. The diluted blood was applied to the membrane, gently mixed, and incubated at 37°C for 1 hour. Hemolysis degree was quantified by measuring the absorbance of the supernatant at 540 nm after centrifugation.

PT and APTT assays were done using a 2 cm × 3 cm electrospun membrane incubated with platelet-poor plasma (PPP) at 37°C for 15 min. The coagulation analyzer automatically measured PT and APTT to assess clotting functionality.

In the partial thromboplastin time (PRT) assay, a 2 × 3 cm electrospun membrane was incubated with PPP at 37°C for 10 min. Subsequently, calcium chloride was added, and the time required for clot formation was recorded.

### 2.8 *In vivo* implantation

Animal procedures were approved by the Animal Welfare Act and Institutional Animal Care and Use Committee at Xi’an Jiaotong University. All rabbits were individually housed in separate cages and provided with *ad libitum* access to diets and water. Ten healthy male New Zealand rabbits, each weighing 2.5 ± 0.5 kg and aged 3 months, were randomly assigned to two groups (*n* = 5 each): mag-AVG, implanted using magnetic anastomosis, and suture-AVG, implanted using conventional manual suturing.

Before surgery, rabbits fasted 12 h and lacked water 6 h. Anesthesia was administered by intravenous injection of 3% pentobarbital sodium at a dosage of 1 mL/kg through the auricular vein. The abdomen was prepared, sterilized and draped. The inferior vena cava was exposed via midline incision. The vena cava was clamped at both ends then excised between clamps.

The procedure for mag-AVG implantation was as follows. First, the magnetic rings were affixed to the vascular clamps separately. Then, the vena cava wall was flipped and wrapped around the rings. Next, one end of the mag-AVG was affixed to the magnetic ring. Heparin saline was injected into the lumen. The other end of the mag-AVG was affixed with the other magnetic ring to complete the anastomosis. Clamps were removed to restore blood flow. For suture-AVG, the vena cava was clamped, transected between clamps, and anastomosed end-to-end with two continuous 8–0 polypropylene sutures. After the operation, both groups of rabbits received analgesia and antibiotics, with no further additional treatments administered.

The status of AVGs was assessed using noninvasive vascular ultrasound (Xuzhou Paier Electronics Co., Ltd.) by vascular medicine specialists on the 2nd and 7th postoperative days. Angiography of the inferior vena cava through the femoral vein was performed on the rabbits at 2 weeks postoperatively.

### 2.9 Statistical methods

Data were analyzed using SPSS 22.0 software (IBM, Armonk, NY). Quantitative data were expressed as mean ± standard deviation ( $\bar{x}\pm \;s$ ). *T* -tests were used for comparisons between two groups, and one-way ANOVA was used for comparisons between multiple groups. The χ2 test was used to compare categorical data. Differences were considered statistically significant at *p* < 0.05.

## 3 Results

### 3.1 Morphological characteristics of electrospun nanofibers

**FIGURE 3:**
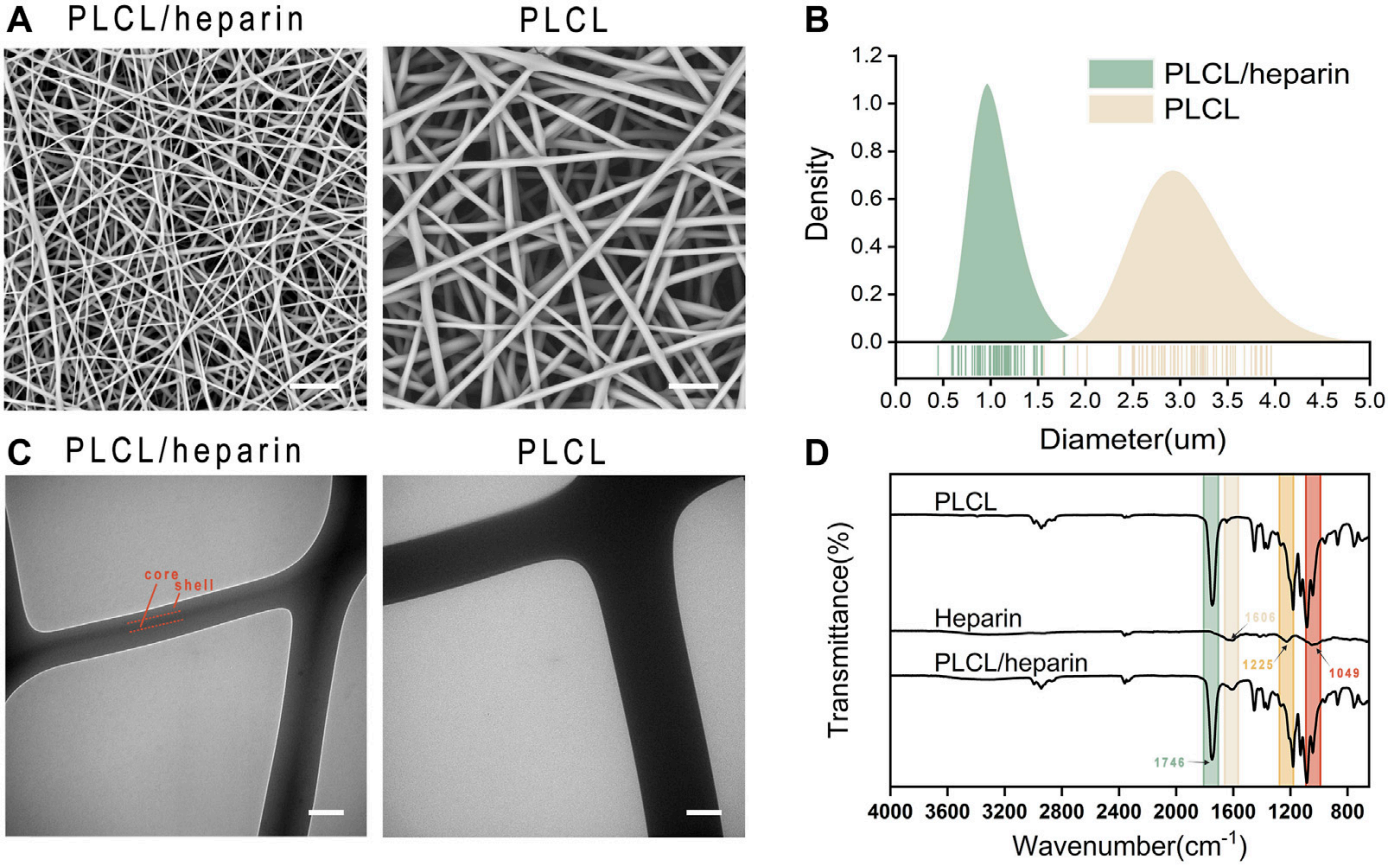
Characterization of electrospun nanofibers. **(A)** SEM images (scale bar = 20 μm); **(B)** Diameter distribution; **(C)** TEM (scale bar = 500 nm); **(D)** FTIR spectra.

The average diameters of the PLCL/heparin and PLCL nanofibers (Figure 3A) were measured to be 1.10 ± 0.22 μm and 2.99 ± 0.56 μm, respectively. It is evident that the PLCL nanofibers displayed a wider range of diameter distribution compared to the PLCL/heparin nanofibers (Figure 3B).

Figure 3C showed a clear contrast between the dark core and the bright shell of PLCL/heparin nanofibers, and the diameter of the core layer constitutes approximately 26.45% of the entire fiber diameter. As shown in Figure 3D, the FTIR spectrum of PLCL nanofibers displayed a strong absorption peak at 1746 cm^−1^ corresponds to the stretching of the C=O bond. Heparin exhibits vibrations at 1,606 cm^−1^ (indicating COO− antisymmetric stretching) and at 1,225 cm^-1^ and 1,049 cm^−1^ (indicating SO_2_- asymmetric stretching). The PLCL/heparin nanofibers revealed a discernible band at 1746 cm^−1^, originating from the -C=O functional group of PLCL, along with peaks at 1,606 cm^−1^, attributable to the -COO− moiety present in heparin. These findings conclusively demonstrate the successful encapsulation of heparin within the PLCL/heparin coaxial structure.

### 3.2 Hemocompatibility assay

**FIGURE 4:**
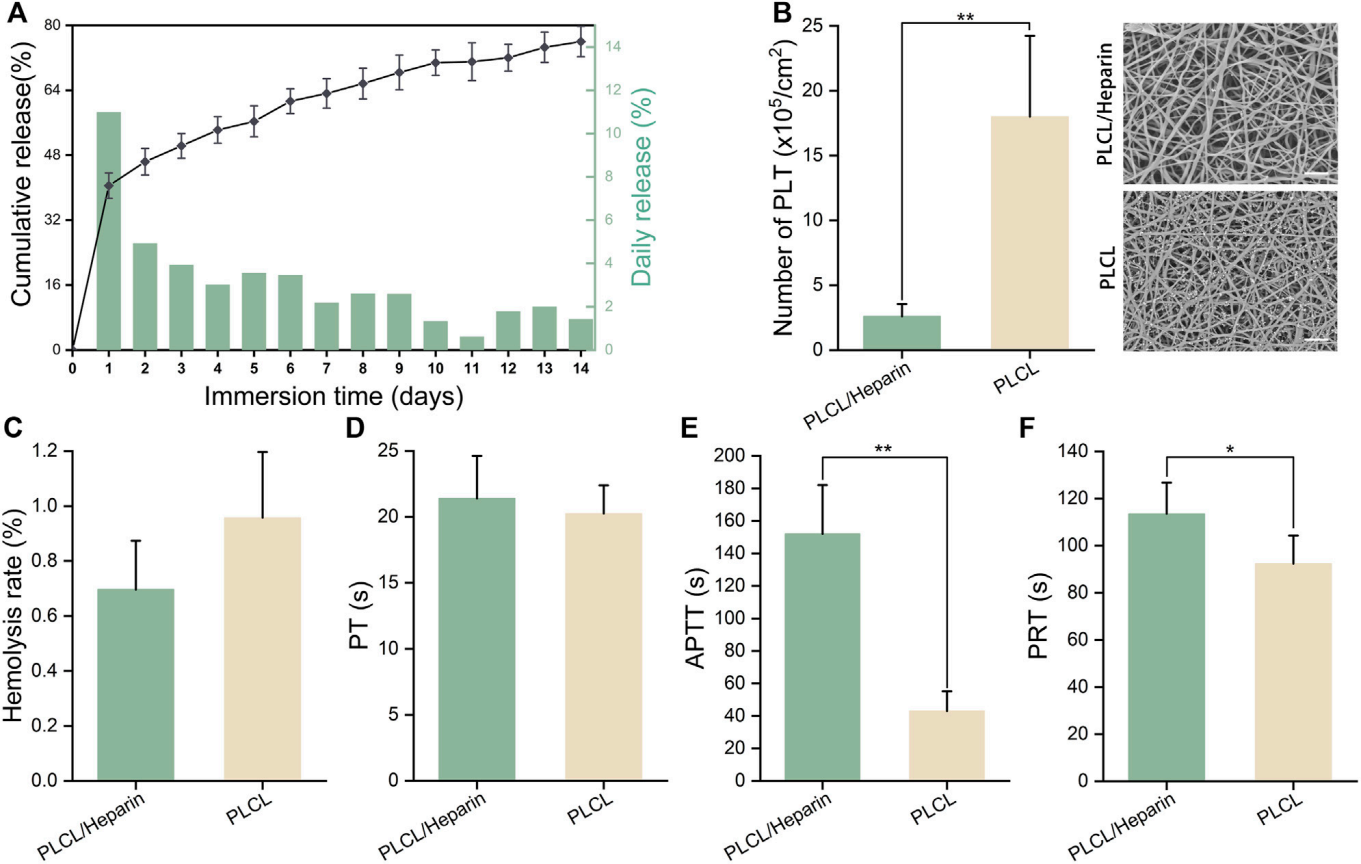
Hemocompatibility of electrospun nanofibers. **(A)** *In vitro* heparin release; **(B)** The number of adherent platelets (scale bar = 20 μm); **(C–F)** Hemolysis rate, prothrombin time (PT), activated partial thromboplastin time (APTT), partial thromboplastin time (PRT).

A distinct two-stage release pattern was observed, characterized by an initial burst release within the first day, followed by a sustained release phase (Figure 4A) (Liu et al., 2022). Specifically, within the initial 24 h, a significant proportion of heparin, amounting to 40.5%, was released. Subsequently, over a span of 14 days, the cumulative amount of released heparin reached approximately 76%.

The SEM images in Figure 4B show that the PLCL nanofibers had a much higher number of adherent platelets than the PLCL/heparin nanofiber group (18.0 ± 6.24 vs. 2.6 ± 0.84 ×10^5^/cm^2^, *p <* 0.01).

The hemolysis rate of both the heparin/PLCL fibers and the PLCL fibers met the international standard ASTM-F756-17, which classifies a hemolytic index of less than 2% as “no hemolysis” (Figure 4C). No significant difference was found in the prothrombin time (PT) between these two groups (20.24 ± 2.15s vs. 21.4 ± 3.21 s, *p =* 0.57) (Figure 4D). However, the activated partial thromboplastin time (APTT) (152 ± 30.0 s vs. 43.2 ± 11.15 s, *p <* 0.01) (Figure 4E) and the partial thromboplastin time (PRT) (116.23 ± 14.21 s vs. 90.01 ± 12.34 s, *p <* 0.01) (Figure 4F) were significantly prolonged in the PLCL/heparin nanofiber group compared to the PLCL nanofiber group.

### 3.3 Mechanical properties of AVGs

**FIGURE 5:**
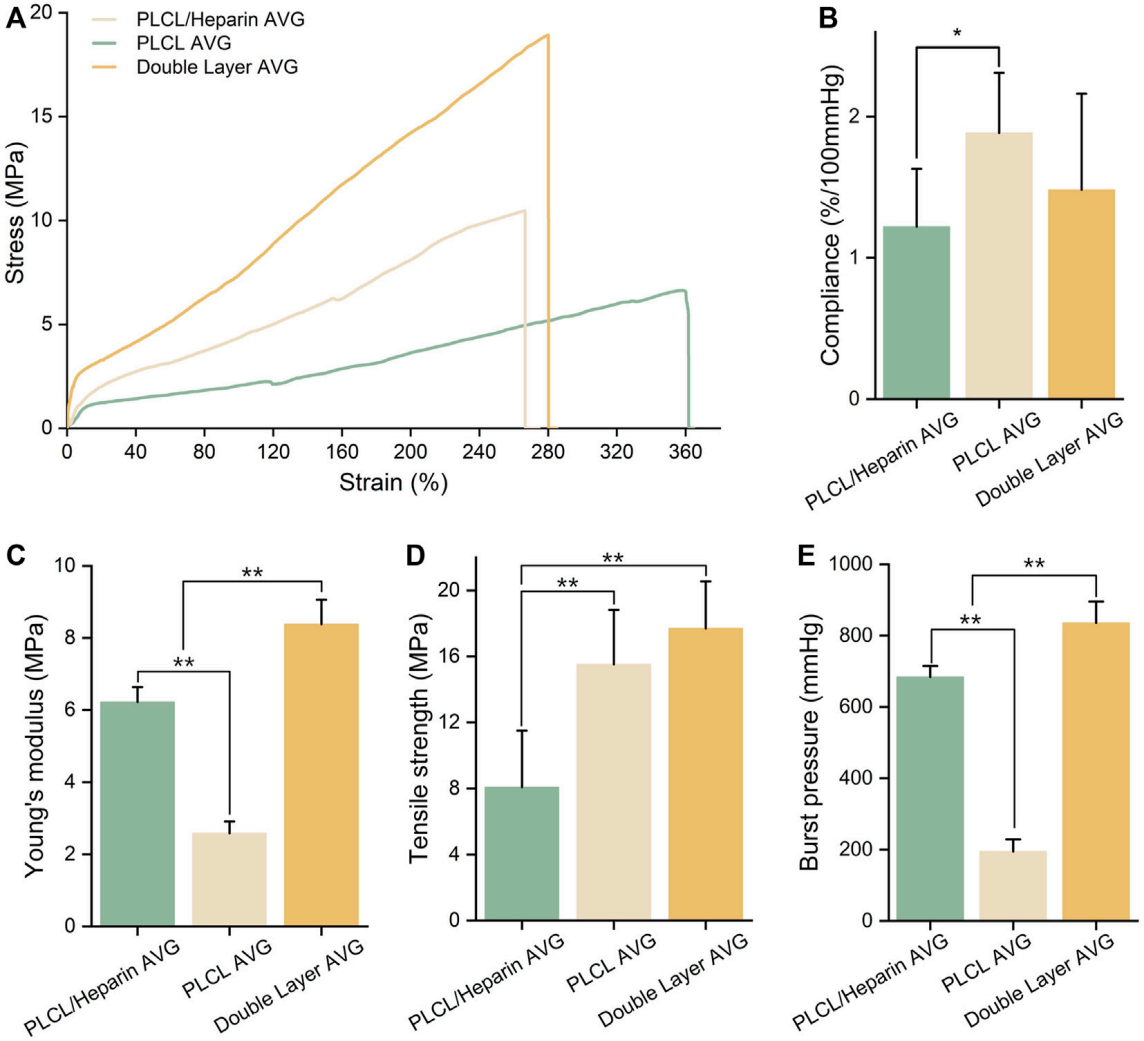
Characterization of AVG. **(A)** Uniaxial tensile stress-strain curves; **(B–E)** Compliance, Young’s modulus, tensile strength and burst pressure of AVG.

The mechanical properties of the AVGs were depicted in Figure 5. The compliance of the AVGs appears to be relatively poor, as observed in Figure 5B (1.22% ± 0.41% vs. 1.88% ± 0.32% vs. 1.48% ± 0.68%/100 mmHg). The compliance of all AVGs was lower than that of the native blood vessels (4.4%/100 mmHg for vein and 11.5%/100 mmHg for artery) (Camasão and Mantovani, 2021). The AVGs constructed using PLCL/heparin nanofibers exhibited significantly higher Young’s modulus (6.22 ± 0.38 vs. 2.58 ± 0.25 MPa, *p <* 0.001), and tensile strength (14.64 ± 2.3 vs. 8.08 ± 3.25 MPa, *p <* 0.01) compared to those made of PLCL nanofibers. Furthermore, the incorporation of a double-layer structure further enhanced the Young’s modulus (8.43 ± 0.81 MPa, *p <* 0.001), while the tensile strength remained largely unaltered (17.52 ± 2.62 MPa, *p =* 0.82). Furthermore, as depicted in Figure 5E, the burst pressure of the PLCL/heparin AVGs exceeded that of the PLCL AVGs but fell short of the double-layer conduits (704.3 ± 31.2 vs. 195.79.07 ± 34.2 vs. 868.21 ± 41.32 mmHg, *p <* 0.001).

### 3.4 *In vivo* implantation of AVGs

**FIGURE 6:**
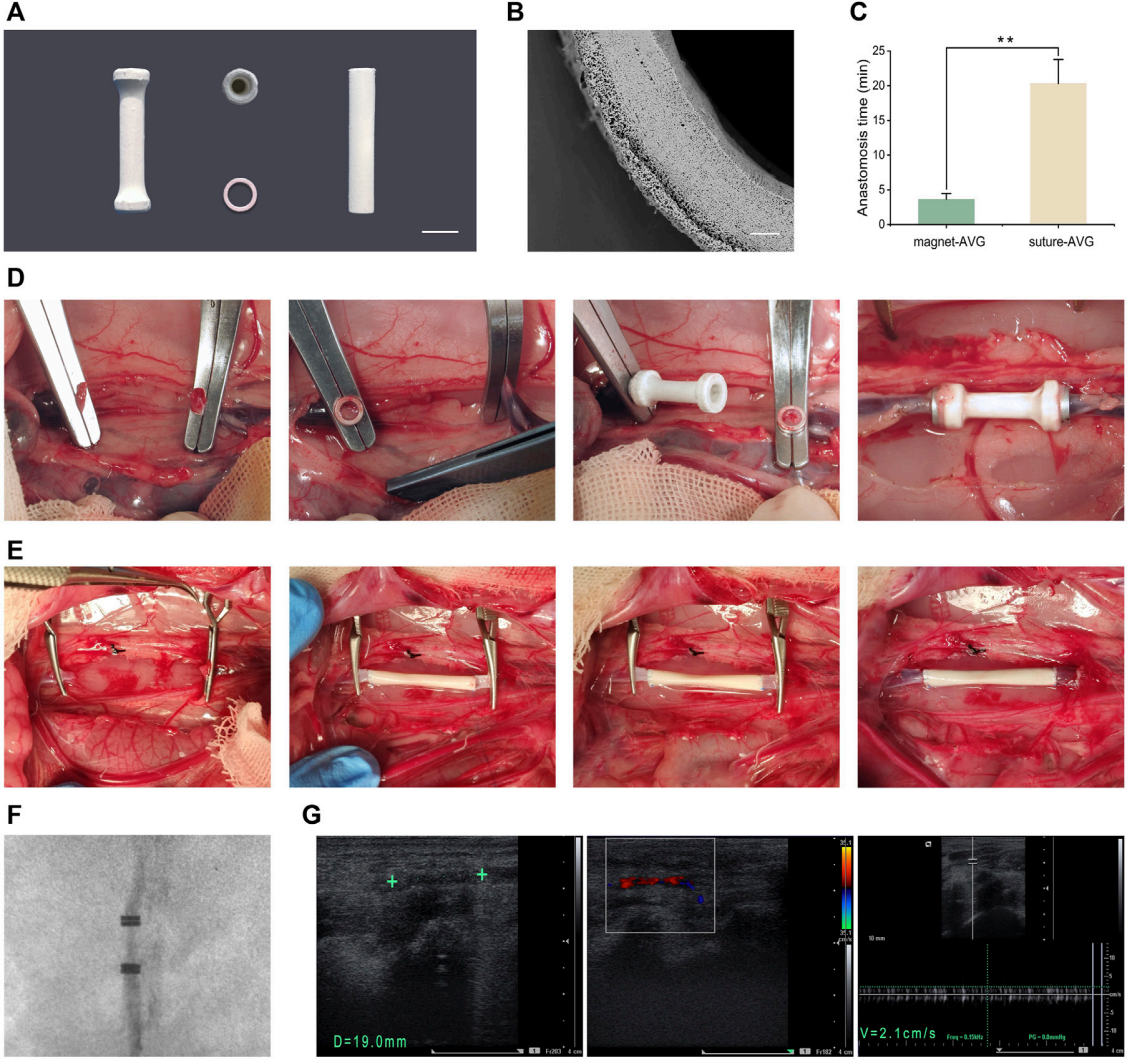
*In vivo* implantation. **(A)** Images of mag-AVG, magnetic rings and suture-AVG; **(B)** The cross-sectional SEM image of AVG; **(C)** Comparison of the anastomosis time between mag-AVG and suture-AVG. **(D)** Images of mag-AVG implantation; **(E)** Images of suture-AVG implantation. **(F)** X-ray image of the implanted mag-AVG; **(G)** Ultrasound images of the implanted mag-AVG on the 2nd day post-implantation; Measurement of AVG length (19 mm) and diameter (3 mm), “+” symbol representing the magnetic rings; Doppler ultrasound reveals blood flow signals within the AVG; The blood flow velocity within the AVG is recorded at 2.1 cm/s.

The AVGs are cylindrical with a 3 mm inner diameter and 20 mm length (Figure 6A). Magnetic rings are embedded at both ends (Figure 6A). The total AVG thickness is ∼0.2 mm with two layers (Figure 6B). The inner PLCL/heparin fiber layer is ∼160 μm thick. The outer PLCL fiber layer is ∼80 μm thick.

To mitigate the challenge of mismatched artificial blood vessels, we selected rabbits with inferior vena cava diameters closely aligned with those of the artificial blood vessels. In cases of slight disparity, we employed techniques such as warm saline or water bag application to dilate the blood vessels. Additionally, we utilized specific methods during manual suturing, such as angulated trimming of the blood vessels, to ensure diameter matching. Procedures of mag-AVG and suture-AVG implantation are illustrated in Figures 6D, E. Magnetic anastomosis was faster, averaging 3.65 ± 0.83 min, compared to suturing, which averaged 20.32 ± 3.45 min (*p <* 0.001, Figure 6C).

All 5 mag-AVG rabbits had successful surgery without complications like infection or bleeding. However, 1 suture-AVG rabbit died during surgery from excessive bleeding at the anastomosis site. For vascular patency, all 5 mag-AVGs were patent on day 2 post-op, 2 remained patent on day 7 (Figure 6F, G), and all were occluded by day 14. In contrast, 2 suture-AVGs showed early occlusion by day 2, and all were occluded by day 7. In summary, the mag-AVGs reduced surgical difficulty and risk and improved early patency, but further optimization of material properties is needed to enhance long-term anticoagulation and patency.

## 4 Discussion

Coaxial electrospinning is a novel technique for producing core-shell nanofibers that provide a robust structure and deliver bioactive agents (Yin et al., 2017; Liu et al., 2020). It has been applied to various fields, such as antibacterial materials (Dong et al., 2023), tissue engineering scaffolds (Iliou et al., 2022; Nagiah et al., 2022), catalysts (Choi et al., 2016), and others. Especially, this technique has unique advantages for creating anticoagulant materials (Huang et al., 2013; Kuang et al., 2018; Strobel et al., 2018). Using a bilayer or multilayer coaxial structure, structural polymers form the outer layer, while natural anticoagulants like heparin are incorporated in the inner layer. This design enables sustained anticoagulant release while maintaining mechanical properties. Compared to physical encapsulation or chemical conjugation, the coaxial structure better preserves anticoagulant bioactivity and provides more controllable release kinetics (Figure 4). Coaxial electrospinning has great potential for small-diameter vascular tissue engineering applications.

However, coaxial electrospinning of AVGs has some limitations. The low elasticity and compliance of electrospun materials impairs suturability and increases the risk of anastomotic complications like leakage and thrombosis (Li et al., 2008a; Rahmati et al., 2020). In this study, for example, the compliance of the AVG was only one-third of natural blood vessels (vein: 4.4%/100 mmHg; artery: 11.5%/100 mmHg) (Camasão and Mantovani, 2021). After suturing, incomplete sealing and enlargement of the anastomosis due to suture cutting led to a high leakage risk. Approaches like cross-linking, coating, blending, and reinforcement have been proposed to improve the compliance (Faturechi et al., 2019b; Furdella et al., 2021), but may also affect hemocompatibility, requiring optimization. Given these limitations, we propose magnetic anastomosis to address the inadequate compliance of electrospun AVGs.

The main finding of this study was that the mag-AVG group had lower surgical complication rates (0% vs. 20%) and higher patency than the suture-AVG group (2 days: 100% vs. 60%; 7 days: 40% vs. 0%). This demonstrates the advantages of magnetic anastomosis compared to sutured anastomosis. First, magnetic anastomosis seals with magnetic fields instead of sutures, resulting in a more effective closure and eliminating leakage risk, as shown in prior intestinal(Chen et al., 2020; Zhang. et al., 2023a), biliary(Jang et al., 2011), vascular(Yang et al., 2018; Zhang et al., 2023b), and biliary-enteric (Li et al., 2008b) anastomoses. Second, magnetic anastomosis leaves no intraluminal sutures and creates a smoother anastomotic stoma, reducing turbulence and platelet adhesion.

Beyond addressing compliance, magnetic anastomosis is easy and quick to perform, with a short learning curve. Brief training enables novice students to outperform experienced surgeons in quality and time (3.65 ± 0.83 vs. 20.32 ± 3.45 min, *p <* 0.001). Magnetic anastomosis also ensures safety and reversibility - unsatisfactory anastomoses can be detached without damage and repeated until favorable.

In comparison to existing AVGs in the market and those documented in the literature, our mag-AVG design offers significant improvements. Traditional AVGs, such as those made from expanded polytetrafluoroethylene (ePTFE) and Dacron, have been associated with high rates of thrombosis and stenosis due to their intrinsic material properties and the technical challenges associated with their implantation (Halbert et al., 2020; Ratner, 2023). These grafts often require precise suturing techniques and can lead to neointimal hyperplasia at the venous anastomosis site. In contrast, our mag-AVG facilitates a rapid and secure connection between vessels with its innovative magnetic mechanism, which not only reduces operative time but also minimizes the inflammatory response typically triggered by suture materials (Deng et al., 2021).

However, our AVGs exhibited lower patency in the long term compared to other studies. For instance, (Wang et al., 2013) attained a 37.5% patency rate over 24 weeks using electrospun coaxial fibers composed of a heparin core and PLCL shell. The low patency rate could be due to several factors. First, unlike other studies, we employed AVGs to replace veins rather than arteries. Veins have lower blood flow velocity and pressure than arteries, increasing thrombus formation risk. Creating artificial veins is challenging and requires outstanding anticoagulation. Second, we abstained from using any anticoagulants in our experiment to assess the optimal effectiveness of our AVGs. Nevertheless, we also recognize the potential benefits of anticoagulant therapy for our AVGs. It is well known that the early stages post-implantation are particularly prone to thrombosis. The administration of anticoagulant medications such as aspirin or heparin during this critical period may potentially enhance the long-term patency of our AVGs. Nonetheless, this hypothesis requires further experimental validation. Third, *in vitro* tests showed ∼76% of heparin released within 2 weeks, impairing long-term anticoagulation. Moreover, long-term patency necessitates fully developed endothelialization, which artificial veins typically lack.

The biocompatibility of the implant is crucial. Our novel graft comprises the biodegradable PLCL and non-degradable magnetic rings, the latter posing dual risks regarding biocompatibility and potential magnetic field effects on tissues. However, several factors in this study mitigate biocompatibility concerns over the short term: Firstly, the electrospun PLCL fibers fully encapsulate the magnetic rings, avoiding any direct exposure. Secondly, we have surface-treated the rings with nickel electroplating, a coating known to enhance biocompatibility by isolating the poorly biotolerated magnetic NdFeB core. Our previous work demonstrates the biocompatibility of this coating (Yang et al., 2018; Wang et al., 2019; Lu et al., 2020). Notably, titanium nitride deposition would be ideal for prolonged implantation according to our expertise, given its widespread application in modifying implantable devices (Iacovacci et al., 2021). Finally, the relatively brief implantation period limits any coating or PLCL degradation, consequently evidencing no tangible biocompatibility issues presently. Nonetheless, regarding potential effects of the magnetic fields, our previous studies found no deleterious effects on surrounding tissue vasculature (Wang et al., 2019; Lu et al., 2020; Xu et al., 2022). Some research even suggests magnetic fields may beneficially promote tissue healing, regeneration and microcirculation (Marycz et al., 2018). Nonetheless, future iterations will ideally incorporate fully degradable magnetic rings composed of nanomaterials that completely degrade within months. This would eliminate any risks of long-term biocompatibility or exposure concerns.

Despite reduced long-term patency, our AVGs have promising applications. Excellent short-term anticoagulation was achieved, with 100% patency by postoperative day 2. Therefore, these AVGs are suitable temporary blood diversion cases. In a separate study, we utilized these AVGs for veno-venous bypass (VVB) during rat liver transplantation (Liu et al., 2022). This enabled rapid bypass without systemic anticoagulation, improving survival and biochemistry. This progress could enhance patient outcomes following complex liver surgeries. Additionally, these vessels may have utility in extracorporeal circulation, ECMO quick connectors, and other scenarios needing swift anastomosis and thrombosis prevention.

However, there exist some limitations in our study. The magnetic ring design requires optimization, as the current thickness is relatively large. Consequently, this method is only suitable for venous reconstruction and cannot yet be utilized for arterial. In future research, we will aim to refine the magnetic ring and AVG design to enable arterial reconstruction applications.

## 5 Conclusion

This study demonstrates the fabrication of small-diameter AVGs using the innovative techniques of coaxial electrospinning and magnetic anastomosis. Coaxial electrospinning enables the incorporation of heparin within the grafts, thereby enhancing their hemocompatibility. Magnetic anastomosis provides a rapid, simple, and safe means of graft implantation while overcoming the compliance mismatch encountered with electrospun grafts. Our findings reveal that grafts implanted by magnetic anastomosis have significantly higher short-term patency and lower surgical risks compared to conventional sutured grafts. However, long-term patency remains a challenge due to the lack of sustained anticoagulation. Further research should optimize the material design and anticoagulation strategies to improve long-term graft performance. Overall, this combinatorial approach of advanced materials and magnetic anastomosis provides a promising platform for small-diameter artificial vascular graft engineering with tremendous clinical potential.

## Data Availability

The raw data supporting the conclusion of this article will be made available by the authors, without undue reservation.
